# Immunization with HIV protease peptides linked to syngeneic erythrocytes

**DOI:** 10.1186/1750-9378-2-9

**Published:** 2007-04-18

**Authors:** Andreas Boberg, Sabrina Dominici, Andreas Brave, Kristian Hallermalm, Jorma Hinkula, Mauro Magnani, Britta Wahren

**Affiliations:** 1 Department of Microbiology, Tumor and Cell Biology, Karolinska Institute, Stockholm, Sweden; 2 Swedish Institute for Infectious Disease Control, Stockholm, Sweden; 3 Institute of Biochemistry "G. Fornaini", University of Urbino, Urbino, Italy; 4 Department for Molecular Virology, IMK, Faculty of Health Science, Linköping University, Linköping, Sweden

## Abstract

New potent vaccine adjuvants are desirable for increasing the efficacy of novel vaccine modalities such as DNA and peptides. We therefore tested if syngeneic erythrocytes could serve as delivery vectors for selected HIV peptides and compared the potency of these constructs to immunization with peptides in phosphate buffered saline or in incomplete Freunds adjuvant.

Immunization of mice with peptides in a low dose (5 ng) coupled to erythrocytes induced a weak immune response in mice. These peptides alone (5 μg) gave no immune responses, while formulating the peptides (50 μg) in IFA induced strong homologous immunity as well as prominent cross reactivity to a related mutant epitope. Thus, vaccine delivery using syngeneic erythrocytes, although attractive for clinical use, might be of limited value due to the low amount of antigen that can be loaded per erythrocyte.

## Findings

Peptide-based vaccines have been shown to be immunogenic in animal models, and well tolerated in man [1,2]. One major benefit of peptide-based immunogens is the ease with which the vaccine can be modulated in order to fit new variants of a variable microbe such as HIV [3]. One application of such a vaccine strategy would be to target viral mutants conferring escape from antiretroviral drugs. As certain known mutations within epitopes of the HIV proteins are associated with resistance to the drugs [4] those epitopes variants could be included in a peptide-based vaccine. However these kinds of vaccines may need to be adjuvanted in order to be used in humans. The strategy of using syngeneic erythrocytes as delivery vectors is attractive, since no external compounds are used. Further, red blood cells (RBCs) are naturally removed by macrophages from the bloodstream and thereby targeted to immune cells. In addition, no toxic side effects have been found in host tissue following RBC-antigen delivery [5]. HIV Tat protein coupled to red blood cells was shown to induce as potent immune response as protein formulated in Freund's adjuvant [6]. The RBC method also induced protective immunity in mice and cats lethally challenged with HSV-1 and FIV-M2, respectively [7,8]. The objective of the present study was to test the efficacy of syngeneic erythrocytes as delivery vectors and adjuvant for peptides deriving from the HIV-1 protease.

A human leukocyte antigen (HLA) A0201 restricted epitope, deriving from HIV-1 protease (PR_75–84 wt_, VLVGPTPVNI) and a mutant variant (PR_75–84 d.mut_) harboring two drug resistance mutations, V82F and I84V were resuspended in phosphate buffered saline (PBS), biotinylated and coupled to syngeneic erythrocytes by avidin-biotin bridges [5,9-12]. The amount of peptide bound to the red blood cells was estimated to 25 ng/mL blood [7,9]. Soluble peptides were formulated in PBS or in incomplete Freunds adjuvant (IFA) to a final concentration of 1 mg/ml. The peptide/IFA mixtures were sonicated (Sonica Vibra Cell VC100, Kenosia Ave, US) at a constant output of 40% for ~30–60 s on ice and the emulsions were used for immunization. C57Bl/6 mice, transgenic for the HLA-A0201 allele, were immunized according to Table 1. The animals were bled 10–12 days following each immunization and three mice per group were sacrificed 10 weeks after the last injection. IFN-γ ELISpot (Mabtech, Nacka, Sweden) was used for readout of cellular immunity. The plates were read by the AID ELISpot reader system. Statistical analysis was performed using GraphPad Prism 4.0. The nonparametric Kruskal-Wallis test was used to identify differences among the different groups, and Mann-Whitney U test was used for post hoc pair-wise comparisons.

**Table 1:** Immunization schedule of the study

**Groups**	**Adjuvant/Carrier^1^**	**Immunogens**	**Amounts**	**Site of injection^1^**	**Symbol used in Figure 1**
PR_75–84 wt_	IFA	VLVGPTPVNI	4 × 50 μg	s.c	**Downward pointing triangle**
PR_75–84 d.mut_	IFA	VLVGPTP**F**N**V**	4 × 50 μg	s.c	**Black filled Diamond**
PR_75–84 d.mut _low^2^	-	VLVGPTP**F**N**V**	4 × 5 μg	s.c	**Blue open Diamond**
RBC-PR_75–84 wt_	RBC	VLVGPTPVNI biotinylated to synergeneic erythrocytes	2 × 5 ng^3 ^+ 2 × 12.5 ng^4^	i.p	**Square**
RBC-PR_75–84 d.mut_	RBC	VLVGPTP**F**N**V **biotinylated to syngeneic erythrocytes	2 × 5 ng^3 ^+ 2 × 12.5 ng^4^	i.p	**Triangle**
RBC_empty_	RBC	Biotinylated syngeneic erythrocytes	3 × 200 μl + 1 × 500 μl blood	i.p	**Circle**
Untreated	-	-	-	-	**Cross**

^1 ^IFA = Incomplete Freunds adjuvant; RBC = Red blood cell; s.c = subcutaneous; i.p = intraperitoneal

^2^A parallel study

^3 ^Corresponds to 200 μl blood

^4 ^Corresponds to 500 μl blood

**Figure 1:**
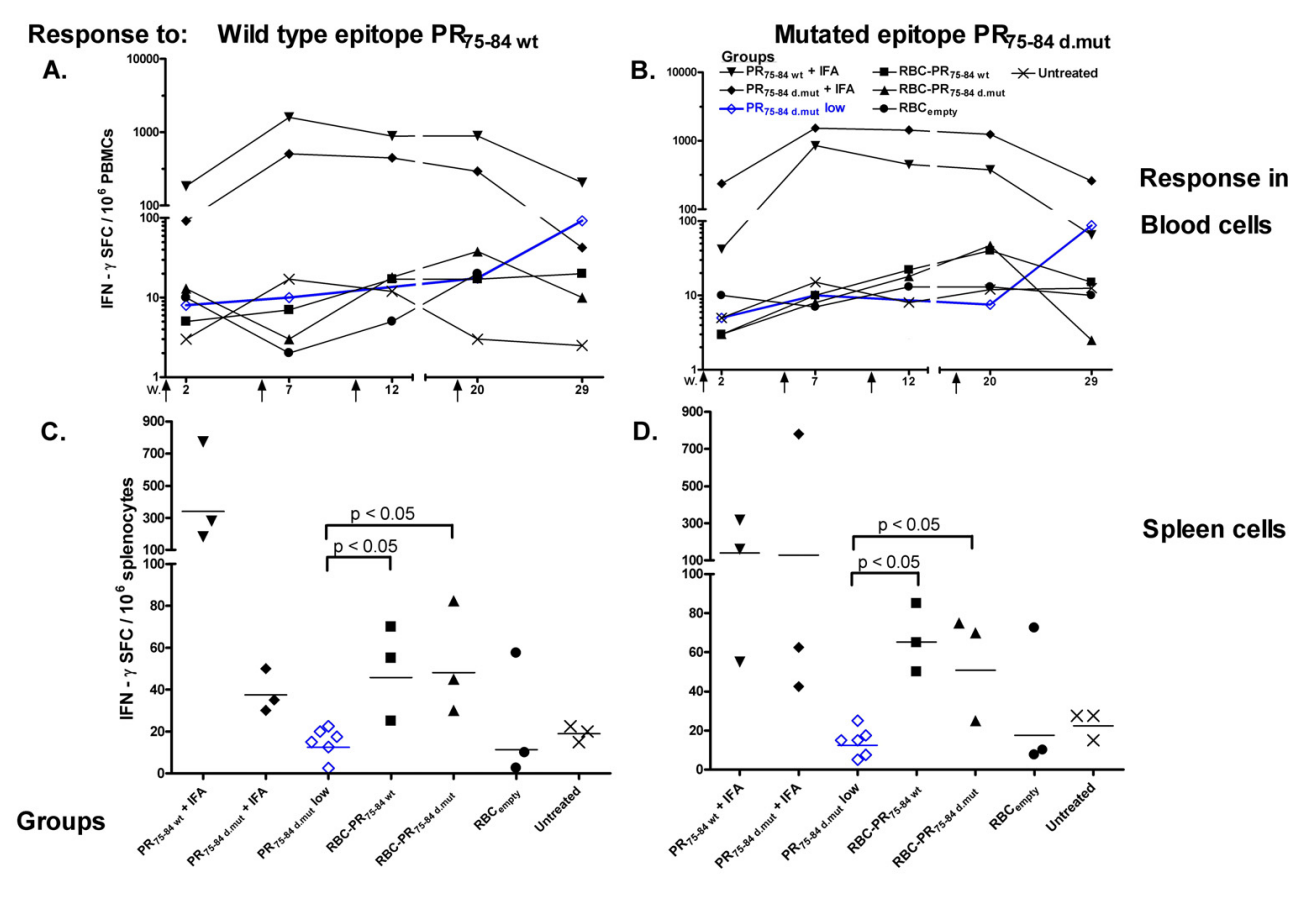
**IFN-γ response to PR_75–84 _epitope variants**. The kinetics of the IFN-γ secretion by peripheral blood mononuclear cells was investigated in response to (A) wild type peptide PR_75–84 wt _and (B) a mutant variant PR_75–84 d.mut_. Vaccination events are indicated by arrows. The response detected in spleens directed against the wild type epitope (C) and the mutant variant (D). Horizontal line indicates the geometric mean response. Blue open diamonds represent the response to peptide from a parallel experiment where mice were immunized three times with 5 μg of the peptide mixed in PBS. Immunizations were performed four weeks apart followed by a boost four months after the third immunization. The animals were bled 10–12 days after each injection.

A strong immune response was detected in blood against the individual peptides PR_75–84 wt _and PR_75–84 d.mut _following peptide/IFA-immunizations, Figures 1A and 1B. This response culminated 2 weeks after the second immunization and 10 weeks after the last injection the response was similar to that seen at week 7, Figures 1A and 1B. Of particular interest is, that immunization with either wild type or mutant epitope induced both high responses to the homologous peptide but also prominent cross-recognition to a related mutant peptide. Ten weeks after the last injection a response to both of the epitope variants could be seen in spleens from the PR_75–84 wt _immunized group, Figures 1C and 1D. The mice, in which RBC was used for peptide delivery showed, after repeated immunization with the RBC-HIV peptide conjugates (5–12,5 ng/injection), only a weak response in spleen cells, Figures 1C and 1D. This response was however significantly stronger (p = 0.0238) than that in mice immunized three times with a five hundred-fold higher concentration of soluble peptide (5 μg) formulated in PBS, Figures 1C and 1D.

We have previously shown that peptide immunization with the wild type protease epitope, PR_75–84 wt_, or the mutant variant, PR_75–84 d.mut_, both formulated in IFA, induces potent immune responses in mice [13]. This suggests that choosing either of these peptides as a component in a vaccine may suppress wild type virus as well as viral variants carrying drug-induced mutations [13]. Based on these findings we compared delivery of the two HIV protease peptides, PR_75–84 wt _and PR_75–84 d.mut_, either prepared in emulsion with IFA or coupled to syngeneic erythrocytes. To further increase the uptake of the peptide/erythrocyte complex by antigen presenting cells, the erythrocytes were chemically modified to be recognized as aging by macrophages and thereby cleared more rapidly by macrophages and antigen-presenting cells [5,10]. A strong immune response was detected after peptide/IFA immunization, whereas only a weak cellular response was measurable upon four consecutive RBC-peptide conjugate immunization. In a parallel experiment, 5 μg of PR_75–84 d.mut _peptide in PBS was used for immunization and this amount of peptide without adjuvant did not induce any immune responses. These findings suggest that erythrocytes may serve as delivery vectors for very small amounts of peptide. Since the loading capacity of erythrocytes is limited, as well as the volume of blood for each injection, the enhanced presentation of peptides to macrophages by the modified erythrocytes is probably not sufficient to compensate for a small amount of peptide antigen in a clinical scenario.

## Competing interests

The author(s) declare that they have no competing interests.

## Authors' contributions

ABo contributed in the design of the study, immunization, read-out and analysis of the immunological data. He also performed the main writing. SD contributed in RBC-peptide linkage and analysis. Further contribution was writing and proof-reading of the manuscript. ABr and KH contributed with data analysis and proof-reading. JH contributed with animal expertise and proof-reading of the manuscript. MM and BW contributed with the project design and proof-reading.
